# mTORC1 function in hippocampal parvalbumin interneurons: regulation of firing and long-term potentiation of intrinsic excitability but not long-term contextual fear memory and context discrimination

**DOI:** 10.1186/s13041-022-00941-8

**Published:** 2022-06-17

**Authors:** Abdessattar Khlaifia, Eve Honoré, Julien Artinian, Isabel Laplante, Jean-Claude Lacaille

**Affiliations:** 1 Department of Neurosciences, Center for Interdisciplinary Research on Brain and Learning (CIRCA) and Research Group On Neural Signaling and Circuitry (GRSNC), Université de Montréal, P.O. Box 6128, Station Downtown, QC H3C 3J7 Montreal, Canada; 2 Present Address: Department of Psychology, University of Toronto Scarborough, ON M1C1A4 Toronto, Canada; 3 Present Address: NeuroService, Neurocentre Magendie , Bordeaux, France

**Keywords:** GABA interneurons, Raptor conditional knock-out mice, Whole-cell recordings, CA1 hippocampus, Contextual fear conditioning

## Abstract

Hippocampal CA1 parvalbumin-expressing interneurons (PV INs) play a central role in controlling principal cell activity and orchestrating network oscillations. PV INs receive excitatory inputs from CA3 Schaffer collaterals and local CA1 pyramidal cells, and they provide perisomatic inhibition. Schaffer collateral excitatory synapses onto PV INs express Hebbian and anti-Hebbian types of long-term potentiation (LTP), as well as elicit LTP of intrinsic excitability (LTP_IE_). LTP_IE_ requires the activation of type 5 metabotropic glutamate receptors (mGluR5) and is mediated by downregulation of potassium channels Kv1.1. It is sensitive to rapamycin and thus may involve activation of the mammalian target of rapamycin complex 1 (mTORC1). LTP_IE_ facilitates PV INs recruitment in CA1 and maintains an excitatory-inhibitory balance. Impaired CA1 PV INs activity or LTP affects network oscillations and memory. However, whether LTP_IE_ in PV INs plays a role in hippocampus-dependent memory remains unknown. Here, we used conditional deletion of the obligatory component of mTORC1, the Regulatory-Associated Protein of mTOR (Raptor), to directly manipulate mTORC1 in PV INs. We found that homozygous, but not heterozygous, conditional knock-out of *Rptor* resulted in a decrease in CA1 PV INs of mTORC1 signaling via its downstream effector S6 phosphorylation assessed by immunofluorescence. In whole-cell recordings from hippocampal slices, repetitive firing of CA1 PV INs was impaired in mice with either homozygous or heterozygous conditional knock-out of *Rptor*. High frequency stimulation of Schaffer collateral inputs that induce LTP_IE_ in PV INs of control mice failed to do so in mice with either heterozygous or homozygous conditional knock-out of *Rptor* in PV INs. At the behavioral level, mice with homozygous or heterozygous conditional knock-out of *Rptor* showed similar long-term contextual fear memory or contextual fear memory discrimination relative to control mice. Thus, mTORC1 activity in CA1 PV INs regulates repetitive firing and LTP_IE_ but not consolidation of long-term contextual fear memory and context discrimination. Our results indicate that mTORC1 plays cell-specific roles in synaptic plasticity of hippocampal inhibitory interneurons that are differentially involved in hippocampus-dependent learning and memory.

## Introduction

Cortical neurons consist of glutamatergic excitatory neurons and GABAergic inhibitory interneurons that represent, respectively, approximately 80% and 20% of the total number [1–4]. Although highly outnumbered, inhibitory interneurons are crucial for normal cortical function by providing a tight control of excitatory neuron activity [1, 2, 5, 6]. Given their importance in gating information flow and sculpting network activity, their dysfunction can result in abnormal brain function and the development of neurological and neuropsychiatric disorders [7, 8].

Inhibitory interneurons display a high diversity at anatomical, neurochemical, transcriptomic and electrophysiological levels [1, 2, 9–11]. In the hippocampus CA1 region, feedforward and feedback inhibition are mediated in part by perisomatic-targeting parvalbumin interneurons (PV INs) and dendritic-targeting somatostatin interneurons (SOM INs) [12–14]. These populations of interneurons are highly dynamic and express multiple types of plasticity at their excitatory input and inhibitory output synapses [14–18]. In addition, PV INs and SOM INs express long-term potentiation of intrinsic excitability (LTP_IE_) and long-term depression of intrinsic excitability (LTD_IE_) respectively [19, 20].

Interestingly, plasticity of intrinsic excitability, which is expressed by a change in action potential firing, is present in both excitatory and inhibitory neurons [20, 21] and plays an important role in memory allocation, consolidation, and updating [21–23]. Mechanistically, plasticity of intrinsic excitability is manifested as changes in action potential threshold, spike accommodation and burst-evoked afterhyperpolarization (AHP), due to alterations in ion channel expression, distribution and function, and which involve several intracellular signaling pathways, like PKA, PKC, CaMKII and mTORC1 [21, 23, 24]. In PV INs, LTP_IE_ is induced by mGluR5 activation that causes a downregulation of Kv1.1 potassium channels, resulting in a sustained increase in PV IN intrinsic excitability [19].

The mechanistic target of rapamycin (mTOR) is a serine/threonine kinase that regulates many aspects of the cell physiology such as cell growth, proliferation and metabolism [25]. mTOR interacts with two structurally and functionally distinct protein complexes, mTOR complex 1 (mTORC1) and 2 (mTORC2). mTORC1 is defined by its specific components Raptor (regulatory-associated protein of mTOR) and PRAS40 (proline-rich Akt substrate 40 kDa), whereas mTORC2 is characterized by Rictor (rapamycin insensitive companion of TOR), the mammalian stress-activated MAP kinase-interacting protein 1 (mSin1), and Protor1 and 2 (protein observed with Rictor 1 and 2) [25]. The two protein complexes display different sensitivity to rapamycin as well as different upstream regulators and downstream targets [25]. mTORC1 activation promotes protein synthesis and cell growth through the regulation of the translational initiation machinery by phosphorylating eukaryotic initiation factor 4e (eIF4E) binding proteins (4EBPs) and p70 S6 kinases (S6K1 and S6K2), whereas mTORC2 activation promotes cell proliferation and survival via Akt [25, 26].

mTORC1, as a key regulator of protein synthesis, plays a cardinal role in long-term synaptic plasticity and memory in excitatory neurons [27]. However, it is also implicated in interneuron synaptic and intrinsic excitability plasticity [19, 27–30]. In CA1 SOM INs, mTORC1 mediates learning-induced LTP at these interneuron input synapses, which in turn regulates CA1 network metaplasticity and hippocampal-dependent contextual fear and spatial memory consolidation [27–29, 31]. In PV INs, mTORC1 may regulate LTP_IE_ since treatment with rapamycin, an inhibitor of mTORC1, impairs LTP_IE_ [19]. Although rapamycin is considered a more effective mTORC1 inhibitor [32], prolonged treatment [33] or higher concentration [34] of rapamycin also inhibits mTORC2. Thus, sensitivity to rapamycin treatment does not necessarily indicate mTORC1 implication.

Interestingly, activity of PV INs and LTP at their excitatory input synapses are critical for CA1 network oscillations and memory consolidation. Following contextual fear conditioning (CFC), PV INs show higher firing coherence with CA1 network oscillations [35]. Moreover, inactivation of PV INs prevents CFC-induced changes in network oscillations and impairs fear memory consolidation [35]. In addition, genetic deletion of γCaMKII in PV INs prevents LTP at Schaffer collateral excitatory synapses onto PV INs and impairs fear memory consolidation [36]. Furthermore, augmented mTORC1 signaling in PV INs impairs contextual fear discrimination [37].

Given the possible role of mTORC1 signaling in plasticity of intrinsic excitability of PV INS and the implication of PV INs in hippocampus-dependent memory, we investigated whether a cell-specific conditional deletion of Raptor, the obligatory component of mTORC1, in PV INs impairs LTP_IE_ in these cells and affects hippocampus-dependent contextual fear memory and context discrimination. Using whole-cell recordings in hippocampal slices, we found that conditional heterozygous and homozygous deletion of *Rptor* in parvalbumin-expressing cells impaired firing and prevented LTP_IE_ in CA1 PV INs. At the behavioral level, mice with conditional heterozygous and homozygous deletion of *Rptor* in parvalbumin-expressing cells showed intact long-term contextual fear memory and context discrimination. Our findings indicate a requirement of mTORC1 activity in PV INs for the normal expression of LTP_IE_ but not for hippocampus-dependent contextual fear memory and context discrimination.

## Materials and methods

### Animals

All animal protocols were in accordance with the Université de Montréal Animal Care Committee (Comité de Déontologie de l'Expérimentation sur les Animaux; CDEA Protocols # 17–001, 17–002, 18–002, 18–003, 19–003, 19–004, 20–001, 20–002, 21–001, 21–002) and experiments were performed in accordance with the Canadian Council of Animal Care guidelines.

Mice with a cell-specific conditional knock-out of *Rptor* in parvalbumin-expressing cells were generated by crossing first, female homozygous *Pvalb*^IRES−Cre^ (The Jackson Laboratory, JAX #008069) with male homozygous *Rptor*^fl/fl^ (JAX #013188). Heterozygous female offsprings *Pvalb*^IRES−Cre/wt^;*Rptor*^wt/fl^ were then crossed with homozygous male *Rptor*^fl/fl^ to generate *Pvalb*^IRES−Cre/wt^;*Rptor*^fl/wt^ (PV-Raptor-Het mice) and *Pvalb*^IRES−Cre/wt^;*Rptor*^fl/fl^ (PV-Raptor-Homo mice) littermates. Homozygous *Pvalb*^IRES−Cre^ mice served as control (PV-Raptor-WT mice). Mice were housed in group of 2–5 per cage with ad libitum access to food and water and maintained under 12 h light/dark cycle, with controlled temperature (~21 °C) and humidity (~55%). All experiments were conducted during the light period. Immunohistochemistry and electrophysiology experiments were carried out on 5 to 11 weeks old male and female mice. For behavioral experiments, 6 to 8 weeks old male mice were used.

### Virus injection

To label PV interneurons in hippocampus, AAV2/9-EF1a-DIO-EYFP (Addgene #27,056; 3.95 × 10^12^ particles/ml) was injected bilaterally in dorsal CA1 hippocampus. 4 to 5 weeks old mice were given an intraperitoneal (IP) injection of ketamine (50 mg/kg i.p.) and xylazine (5 mg/kg i.p.) and placed in a stereotaxic frame (Stoelting). Viral solution (0.8µL) of AAV2/9-EF1a-DIO-EYFP was injected using a 10 μl Hamilton syringe (coordinates relative to bregma: AP -2.46 mm; L ± 1.75 mm; DV -1.5 mm). The needle was left in place for 5 min after injection. Whole-cell patch-clamp recording experiments were performed between 7 to 15 days after AAV injection to allow animals recovery and EYFP expression.

### Immunohistochemistry

Mice were anaesthetized with sodium pentobarbital (MTC Pharmaceuticals, Cambridge, Ontario, Canada) and transcardially perfused with ice-cold 0.1 M phosphate buffer (PB) and then with 4% paraformaldehyde in 0.1 M PB. Brains were postfixed overnight and then cryopreserved in 30% sucrose. Coronal brain sections were obtained with a freezing microtome (Leica SM200R, Germany) at 50 µm thickness. Membrane permeabilization was performed by incubating sections in 0.3–0.5% Triton X-100 in 0.01 M saline PB (PBS) for 15 min. Unspecific binding was blocked by incubating sections for 1 h in 10% normal goat serum in 0.1% Triton X-100/PBS. Sections were incubated in primary antibody for 48 h at 4 °C (Mouse monoclonal Raptor; 1/500; Millipore catalog #05–1470, Burlington, MA, RRID:AB_11212192). Sections were then washed in PBS and incubated with secondary antibody (Rhodamine-conjugated goat anti-mouse IgG1; 1/200; Jackson Immunoresearch Laboratories, West Grove, PA) for 90 min at room temperature. Sections were washed in PBS before mounting, coverslipped with ProLong™ Diamond (Life technologies) and examined using a Nikon microscope (Nikon Eclipse E600; Nikon, Japan) equipped with epifluorescence. Images were acquired with the Simple PCI software (CImaging Systems, Compix Inc., PA). The number of EYFP-positive interneurons in CA1 with colocalization of Raptor immunofluorescence were counted and expressed as percentage of the total EYFP-positive cells per sections. A total of 3 animals per group coming from 3 independent experiments were analyzed (2–4 sections/animal for a total of 97 cells in PV-Raptor-WT, 61 cells in PV-Raptor-Het, and 89 cells in PV-Raptor-Homo mice).

### S6 phosphorylation immunofluorescence

Brain sections were prepared as described above for EYFP-positive parvalbumin interneuron visualization and immunostaining for phospho-S6^S240/244^ was performed. Individualized free-floating sections were permeabilized and treated for unspecific binding as described above. Slices were then incubated with rabbit monoclonal phospho-S6 antibody (1/1000; anti-phospho-S6^S240/244^; Cell Signaling catalog #5364, Beverly, MA, RRID:AB 10,694,233) for 48 h at 4 °C, and subsequently with Alexa Fluor 594-conjugated goat anti-rabbit IgGs (1/500; Jackson Immunoresearch Laboratories) for 90 min at room temperature. Images were acquired with a Zeiss LSM 880 (Carl Zeiss, Oberkochen, Germany) confocal microscope at excitation 488 and 543 nm. Images in wild-type and conditional knock-out mice were acquired using the same parameters. Phospho-S6 cell fluorescence was quantified using ImageJ software (National Institute of Health; https://github.com/imagej/imagej1) by comparing density in cells corrected for background. Cell fluorescence was measured typically in 9–28 field of views from 3–4 sections per animal and was averaged per animal. A total of 5 animals per group coming from 5 independent experiments were analyzed (total of 441 cells in PV-Raptor-WT; 362 cells PV-Raptor-Het; 444 cells in PV-Raptor-Homo mice).

### Slice preparation and electrophysiology

Hippocampal slices (300 µm thickness) were prepared from PV-Raptor-Het, PV-Raptor-Homo and PV-Raptor-WT mice. Mice were anesthetized with isoflurane and the brain was quickly removed and placed in ice-cold oxygenated (95% O_2_, 5% CO_2_) sucrose-based cutting solution containing the following (in mM): 87 mM NaCl, 2.5 mM KCl, 1.25 mM NaH_2_PO_4_, 7 mM MgSO_4_, 25 mM NaHCO_3_, 25 mM D-glucose, 75 mM sucrose, 1 mM ascorbic acid, 3 mM pyruvic acid and 0.5 mM CaCl_2_. Hippocampal slices were obtained using a vibratome (Leica, VT1000S) and transferred to oxygenated artificial cerebrospinal fluid (aCSF) containing the following (in mM): 124 mM NaCl, 5 mM KCl, 1.25 mM NaH_2_PO_4_, 2 mM MgSO_4_, 2 mM CaCl_2_, 26 mM NaHCO_3_ and 10 mM dextrose (pH = 7.3–7.4; 295–300 mOsmol/L) for 30 min at 30 °C. Slices were left in aCSF at room temperature for an additional 30 min. Individual hippocampal slices were then placed in a submerged recording chamber on the stage of an upright microscope (Nikon Eclipse, E600FN), equipped with a water immersion long-working distance objective (× 40, Nomarski optics) and an infrared video camera. Slices were perfused at 2.5 mL/min with aCSF at 30 °C. Whole-cell current-clamp recordings were obtained from identified EYFP-expressing PV interneurons located in or at the border of CA1 stratum pyramidale. Patch glass electrodes (3–5 MΩ) were filled with internal solution containing the following (in mM): 120 K-gluconate, 10 KCl, 0.5 EGTA, 10 HEPES, 2.5 MgATP, 0.3 NaGTP, 10 Na_2_-phosphocreatine, 0.1 spermine, pH 7.3–7.4, and 280 ± 10 mOsmol. Recordings were performed using Multiclamp 700A/B amplifier (Molecular Devices) and digitized using Digidata 1440A and pClamp 10 software (Molecular Devices). Signals were filtered at 2 kHz, digitized at 10 kHz and stored on a PC. Recordings were included if the series resistance varied by < 20% and if the holding current was stable.

Membrane properties were recorded in current-clamp mode at a holding potential of -60 mV. Resting membrane potential (RMP) was directly measured after rupturing the cell membrane at a holding current I = 0 pA. Input resistance (Rin) was calculated using a linear regression of voltage deflections (± 15 mV) in response to current steps (800 ms, 20 pA increment, holding membrane potential -60 mV). Action potential (AP) amplitude was measured as the difference in membrane potential between the threshold and the peak. The time difference between the current pulse onset and the AP peak was defined as AP latency. Action potential threshold was taken as the first voltage point at which the slope of the membrane potential exceeded 20 mV/ms. AP half-width was measured as action potential duration at half-amplitude. Fast-afterhyperpolarization (fAHP) amplitude was measured as the difference between AP threshold and the negative voltage peak after the AP. PV interneuron firing rate was evaluated using a series of depolarizing current pulses (800 ms duration) from 100 to 440 pA with increments of 20 pA.

Long-term potentiation of intrinsic excitability was induced in the presence of the GABA_A_ channel blocker picrotoxin (100 µM) by high frequency stimulation (HFS) consisting of 10 bursts of 10 stimulations delivered at 100 Hz every 3 s through a theta-glass stimulating electrode filled with aCSF and placed in stratum radiatum.

### Contextual fear conditioning

Mice were handled for 3 days prior to fear conditioning experiments to familiarize them with the experimenter, room and procedures. Mice were trained in conditioning chambers that were housed in sound- and light-isolated cubicles (Coulbourn Instruments, Withehall, PA). The chambers were made of a stainless-steel grid floor, overhead LED lighting, camera and supplied with background noise (60 dB) by an air extractor fan. The experimental protocol was based on Artinian and coworkers [29]. The training context was rectangular with 2 transparent walls and 2 stainless-steel walls and was cleaned with 70% ethanol before and after each trial. For context discrimination, the neutral context was triangular with transparent walls. For conditioning, mice were placed in the conditioning chamber, allowed to freely explore for 2.5 min, and then received 5 presentations of unconditioned stimuli (1 s foot shock, 0.8 mA). To test for long-term contextual fear memory, mice were returned to the training context 24 h after conditioning during a test period of 2.5 min. To test for contextual discrimination, 5 h after the test in the training context on the memory test day, a sub-group of mice were allowed to explore the new neutral context for 2.5 min. Freezing behavior was assessed using FreezeFrame (Coulbourn Instruments). Discrimination ratio was calculated as the amount of freezing in (training context)/(training context + neutral context) [29]. A ratio of 1 indicates that mice were able to discriminate the contexts perfectly, and a ratio of 0.5 means that they were unable to discriminate.

### Statistical analysis

Data were expressed as mean ± SEM and analyzed using GraphPad Prism 8 (GraphPad Software Inc.). Data were tested for normality using Shapiro–Wilk test and equal variance with Kolmogorov–Smirnov. For within-group comparisons, paired t-test and Wilcoxon signed-rank test were used for normally and non-normally distributed distributed data, respectively. For multiple comparisons, one-way ANOVA and two-way ANOVA were used, followed by Tukey’s post hoc test.

## Results

### Conditional knock-out of *Rptor* in PV interneurons causes a deficit in mTORC1 signaling

**Fig. 1:**
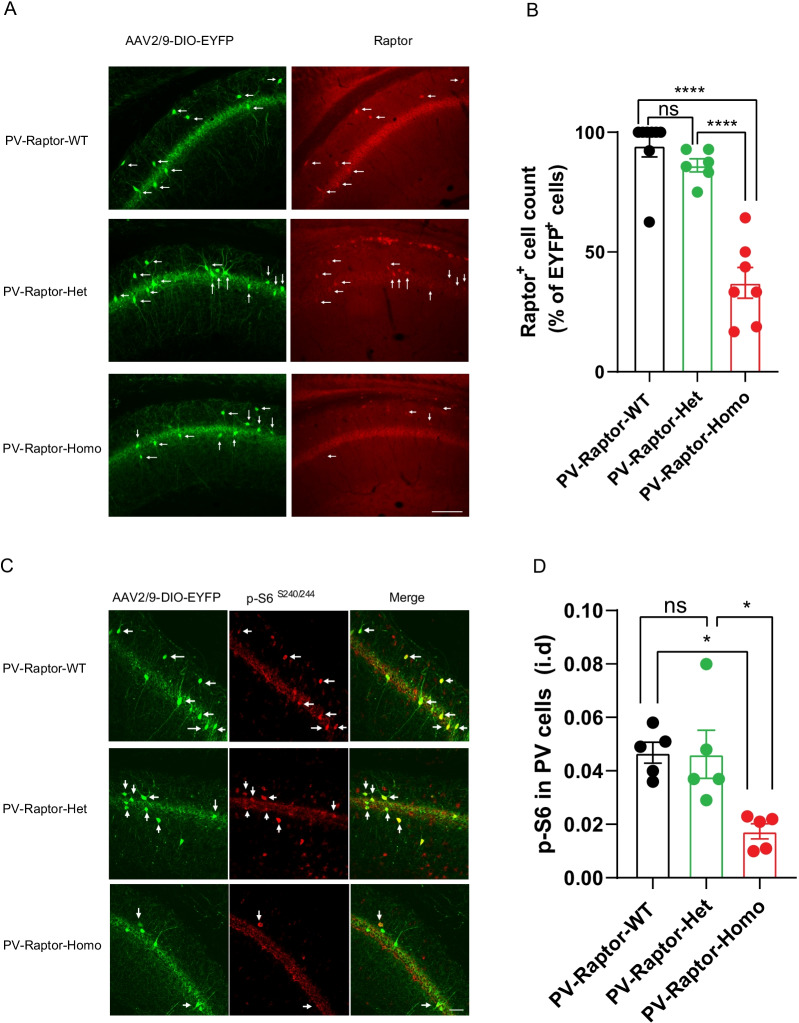
Homozygous conditional knock-out of *Rptor* in PV interneurons causes a deficit in mTORC1 activity. **A** Left, representative images of EYFP-positive PV interneurons (white arrows) in PV-Raptor-WT, PV-Raptor-Het and PV-Raptor-Homo mice injected with AAV2/9-DIO-EYFP in dorsal CA1 hippocampus. Right, representative images of Raptor-positive (red) EYFP-expressing PV interneurons (white arrows, co-labeling) in PV-Raptor-WT, PV-Raptor-Het and PV-Raptor-Homo mice. Scale bar 100 µm. **B** Summary graph showing reduced percentage of EYFP-positive cells that are also Raptor-positive in PV-Raptor-Homo mice relative to PV-Raptor-Het and PV-Raptor-WT mice (n = 7 sections from 3 PV-Raptor-WT mice, 6 sections from 3 PV-Raptor-Het mice, and 7 sections from 3 PV-Raptor-Homo mice, from 3 independent experiments). **C** Representative images showing EYFP-positive cells (green), S6^S240/244^ phosphorylation (red) and co-labeling (merged) in CA1 hippocampus of PV-Raptor-WT, PV-Raptor-Het and PV-Raptor-Homo mice. **D** Summary graph showing reduced p-S6 immunofluorescence in CA1 PV interneurons of PV-Raptor-Homo mice relative to PV-Raptor-Het and PV-Raptor-WT mice (n = 5 mice/group from 5 independent experiments, scale bar 50 µm). **** *p* < 0.0001, * *p* < 0.05, ns not significant

First, we verified that deleting *Rptor* specifically in PV cells affects Raptor expression in hippocampal CA1 PV interneurons. We quantified the number of EYFP-expressing hippocampal CA1 PV INs that are immunopositive for Raptor in PV-Raptor-WT, PV-Raptor-Het and PV-Raptor-Homo mice. We found that the number of PV INs expressing EYFP and immunopositive for Raptor are significantly reduced in PV-Raptor-Homo mice relative to PV-Raptor-WT mice (PV-Raptor-WT n = 8 sections, PV-Raptor-Het n = 6 sections, PV-Raptor-Homo n = 7 sections, for 3 mice in each group; One Way ANOVA, F_(2, 18)_ = 4 p < 0.0001, Tukey’s multiple comparisons test, PV-Raptor-WT vs PV-Raptor-Het, p = 0.5; PV-Raptor-WT vs PV-Raptor-Homo, p < 0.0001; PV-Raptor-Het vs PV-Raptor-Homo, p < 0.0001; Fig. 1A, B). PV-Raptor-Het mice failed to show such a decrease in number of PV INs expressing EYFP and immunopositive for Raptor. Thus, Raptor expression is impaired in hippocampal PV INs of PV-Raptor-Homo mice.

Next, we examined the effects of conditional knock-out of *Rptor* on mTORC1 activity by assessing phosphorylation of ribosomal protein S6^S240/244^ (p-S6), a downstream effector of mTORC1, in CA1 PV INs using immunofluorescence. The level of p-S6 was reduced in EYFP-expressing CA1 PV INs of PV-Raptor-Homo mice relative to PV-Raptor-WT or PV-Raptor-Het mice (n = 5 mice in each group; one-way ANOVA, F (2, 12) = 8, p = 0.006; Tukey’s multiple comparisons test, PV-Raptor-WT vs PV-Raptor-Het, p = 0.99; PV-Raptor-WT vs PV-Raptor-Homo, p = 0.01; PV-Raptor-Het vs PV-Raptor-Homo, p = 0.01; Fig. 1C, D). The level of p-S6 was unaffected in CA1 PV INs of PV-Raptor-Het mice relative to PV-Raptor-WT mice. These results confirm that mTORC1 signaling, as assessed by p-S6, is impaired in hippocampal PV INs of PV-Raptor-Homo mice.

### Conditional *Rptor* knock-out in PV interneurons impairs repetitive firing

**Fig. 2:**
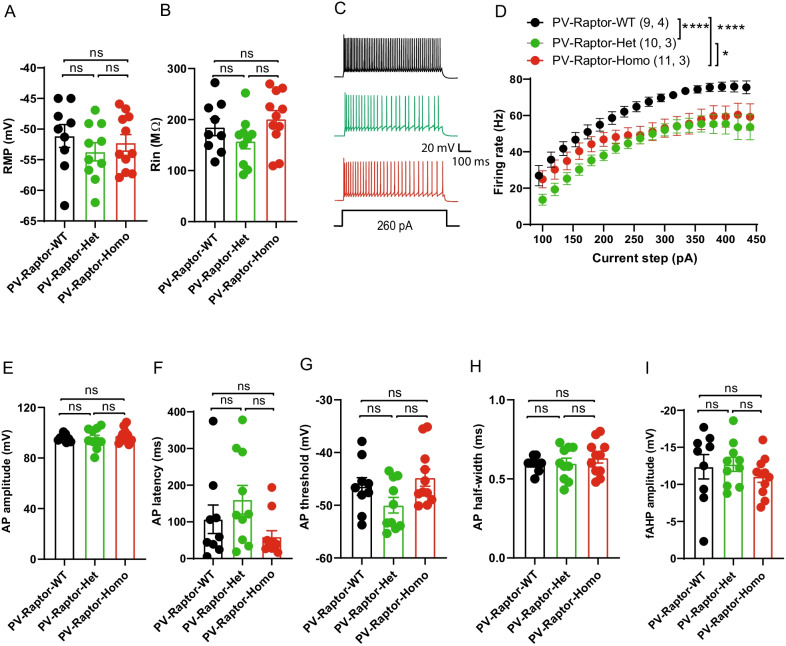
Conditional knock-out of *Rptor* in PV interneurons impairs firing properties. **A** and** B** Summary bar graphs showing intact: resting membrane potential (**A**) and input resistance (**B**) of CA1 PV interneurons from PV-Raptor-Het (green) and PV-Raptor-Homo (red) relative to PV-Raptor-WT mice (black). **C** Representative voltage responses of PV interneurons in response to a somatic depolarization (260 pA), illustrating the decrease in evoked firing of PV cells from PV-Raptor-Het and PV-Raptor-Homo mice relative to PV-Raptor-WT mice. **D** Summary plot of frequency-current relationship for all cells showing reduced evoked firing of PV interneurons from PV-Raptor-Homo (n = 11 cells, 3 mice) and PV-Raptor-Het (n = 10 cells, 3 mice) mice relative to PV-Raptor-WT mice (n = 9 cells, 4 mice). **E**–**I** Summary bar graphs showing intact: action potential amplitude (**E**), action potential latency (**F**), action potential threshold (**G**), action potential half-width (**H**) and fast after-hyperpolarization amplitude (**I**) of CA1 PV interneurons from PV-Raptor-Het (green) and PV-Raptor-Homo (red) relative to PV-Raptor-WT mice (black). * p < 0.05, **** p < 0.0001, ns not significant

Activation of mTORC1 is generally linked to stimulation of protein synthesis [27, 38, 39]. However, mTORC1 activation also represses the synthesis of specific mRNAs, such as the Kv1.1 channel, a voltage-gated potassium channel that regulates neuronal excitability [40, 41]. Therefore, we determined if conditional *Rptor* knock-out in PV INs affected their membrane and firing properties. Whole-cell patch-clamp recordings were obtained from EYFP-expressing PV INs located in or near the CA1 *stratum pyramidale* in acute slices from control and PV conditional *Rptor* knock-out mice (PV-Raptor-WT n = 9 cells in 4 mice, PV-Raptor-Het n = 10 cells in 3 mice, and PV-Raptor-Homo n = 11 cells in 3 mice). We found that PV INs from control and conditional knock-out mice had similar resting membrane potential (One Way ANOVA, F (2, 27) = 0.65 p = 0.52; Fig. 2A) and input resistance (One Way ANOVA, F(2, 27) = 1.93 p = 0.16; Fig. 2B), suggesting intact basic membrane properties.

Then, we assessed PV interneurons repetitive firing properties in response to somatic depolarizations. PV INs from control and mutant mice responded to incremental somatic depolarization with increasing number of action potentials (Fig. 2C, D). However, PV INs from PV-Raptor-Het and PV-Raptor-Homo mutant mice fired less action potentials compared to those from PV-Raptor-WT mice (Two Way ANOVA, F (2, 27) = 7.73 p = 0.002; Tukey’s multiple comparisons tests, PV-Raptor-WT vs. PV-Raptor-Het p < 0.0001, PV-Raptor-WT vs PV-Raptor-Homo p < 0.0001, PV-Raptor-Het vs. PV-Raptor-Homo p = 0.012; Fig. 2C, D). The impairment in firing was greater in PV INs from PV-Raptor-Het mice compared to those from PV-Raptor-Homo mice. These results suggest that conditional hetero- and homozygous knock-out of *Rptor* in PV interneurons have impaired firing output.

Next, we examined whether the firing impairment of PV INs could be explained by changes in action potential properties. We found that PV INs from PV-Raptor-Het and PV-Raptor-Homo mice display similar action potential amplitude (One Way ANOVA, F(2, 27) = 0.44 p = 0.65, Fig. 2E), latency to first action potential (One Way ANOVA, F(2, 27) = 2.60 p = 0.09 Fig. 2F), action potential threshold (One Way ANOVA, F(2, 27) = 02.89 p = 0.07, Fig. 2G), action potential half-width (One Way ANOVA, F(2, 27) = 0.54 p = 0.58, Fig. 2H) and fast afterhyperpolarization amplitude (One Way ANOVA, F(2, 27) = 0.64 p = 0.53, Fig. 2I). Together, these data indicate that conditional hetero- and homozygous knock-out of *Rptor* in PV interneurons impairs their repetitive firing properties without affecting their resting membrane potential, input resistance and action potential properties.

### Conditional *Rptor* knock-out in PV interneurons impairs long-term potentiation of intrinsic excitability

Hippocampal GABAergic interneurons are highly dynamic and display several forms of long-term plasticity of synapses and intrinsic excitability [14, 15, 19–21, 42]. CA1 parvalbumin-expressing basket cells show long-term potentiation of intrinsic excitability (LTP_IE_) via mGluR5 activation and down-regulation of Kv1.1 channels, which is prevented by rapamycin, an inhibitor of mTORC1 [19]. Given that Raptor is obligatory for mTORC1 function, we tested if conditional knock-out of *Rptor* in PV INs could affect LTP_IE_. We obtained whole-cell patch-clamp recording from EYFP-positive PV interneurons located in or near CA1 *stratum pyramidale*. After establishing their fast-spiking phenotype in current-clamp mode via the injection of depolarizing currents, we adjusted (i) the intracellular depolarizing current to evoke approximately 5 action potentials, and (ii) the extracellular electrode simulation in *stratum radiatum* to elicit an EPSP of approximately 2 mV in amplitude [19]. After obtaining a stable baseline (5 min) of depolarization-evoked firing, we applied a high frequency stimulation (HFS) to the Schaffer collaterals pathway that consisted of 10 pulses at 100 Hz, repeated 10 times at the frequency of 3 Hz, and recorded PV interneuron spiking induced by the same somatic depolarization for up to 30 min [19].

**Fig. 3:**
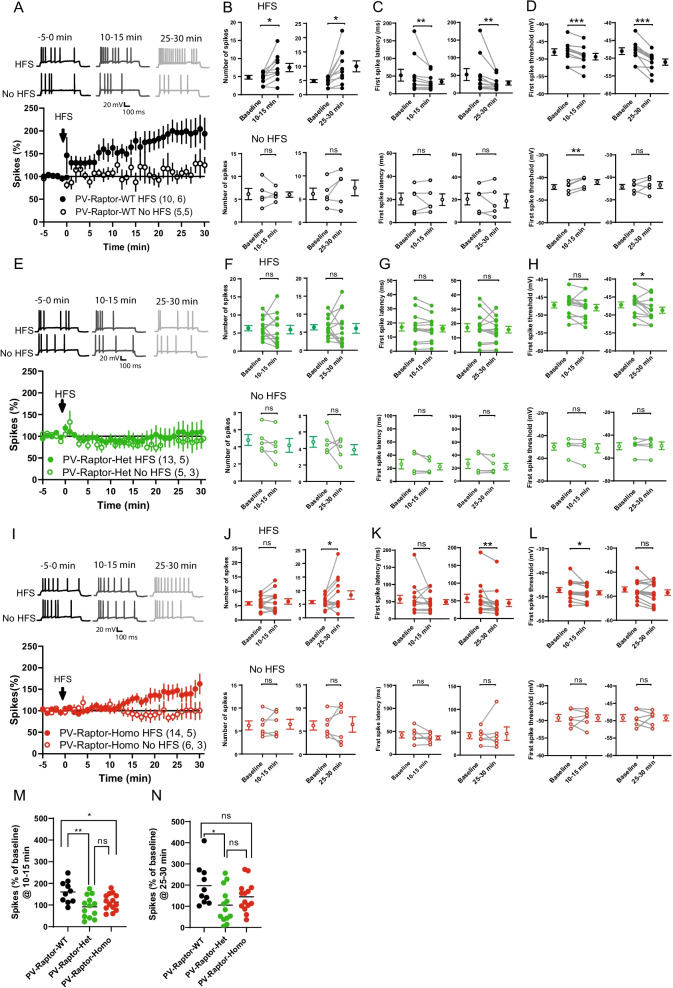
Conditional knock-out of *Rptor* in PV interneurons impairs LTP_IE_. **A** Representative traces (top) and time plot for all cells (bottom) of depolarization evoked firing showing long-lasting increase of firing in the group receiving HFS (filled circles, HFS) but not in the non-tetanized control group (open circles, No HFS) in CA1 PV interneuron from PV-Raptor-WT mice. **B-D** Summary plots of spikes number (**B**), latency to first spike (**C**) and first spike threshold (**D**) measured at -5 to 0 min baseline *versus* 10–15 min (left), or 25–30 min (right) post-HFS in cells of the tetanized group (HFS, top) and control group (No HFS, bottom). Individual data points before and after are joined by lines; means ± sem are indicated to the side for each group. **E**–**H** Similar data representation showing absence of long-lasting potentiation of intrinsic excitability at 10–15 min and 25–30 min after HFS in PV interneurons from PV-Raptor-Het mice. **I**–**L** Similar data representation showing a block of long-lasting potentiation of intrinsic excitability at 10–15 min, but not at 25–30 min, after HFS in PV interneurons from PV-Raptor-Homo mice. **M** and **N** Summary plots of spike increases relative to baseline for all cells measured at 10–15 min (**M**) and 25–30 min (**N**) after HFS in PV interneurons, showing block of LTP_IE_ at 10–15 and 25–30 min after HFS in PV-Raptor-Het mice, and at 10–15 min post HFS in PV-Raptor-Homo mice, relative to PV-Raptor-WT mice. *** p < 0.001, ** p < 0.01, * p < 0.05, ns not significant

As previously reported in rat [19], we found that in PV INs from PV-Raptor-WT mice (n = 10 cells in 6 mice), HFS of Schaffer collaterals resulted in long-lasting potentiation of PV IN evoked firing (159.8% ± 15.8% of baseline at 10–15 min and 197.3% ± 3.3% of baseline at 25–30 min post-HFS, paired t-tests, p = 0.013 and p = 0.011 respectively, Fig. 3A, B). In the absence of HFS of Schaffer collateral pathway, we observed no change in evoked firing of PV INs (n = 5 cells from 5 mice, 105.18% ± 14% of baseline at 10–15 min and 118.65% ± 15% of baseline at 25–30 min post-HFS, paired t-tests, p = 0.88 and p = 0.21 respectively, Fig. 3A, B). LTP_IE_ was associated with a reduction in the latency of the first action potential (79.89 ± 5.49% of baseline at 10–15 min post-HFS and 68.17 ± 6.67% of baseline at 25–30 min post-HFS, Wilcoxon tests, p = 0.004 and 0.004 respectively, Fig. 3C) and a hyperpolarization of the first action potential threshold (102.8 ± 0.54% of baseline at 10–15 min post-HFS and 106.54 ± 1.16% of baseline at 25–30 min post-HFS, paired t-tests, p = 0.0007 and 0.0003 respectively, Fig. 3D). In the absence of HFS of Schaffer collateral pathway, no change was observed in the first action potential latency (97.13 ± 14.73% of baseline at 10–15 min post-HFS and 91.8 ± 15.23% of baseline at 25–30 min post-HFS, paired t-tests, p = 0.83 and 0.55 respectively, Fig. 3C), and only a depolarization of the first action potential threshold was seen at 10–15 min post-HFS (95.04 ± 0.7% of baseline at 10–15 min post-HFS and 98.2 ± 2.76% of baseline at 25–30 min post-HFS, paired t-tests, p = 0.0018 and 0.55 respectively, Fig. 3D). The reduction in the first spike latency and threshold in the HFS group but not in the No HFS group is consistent with a modulation of Kv1.1 during LTP_IE_ in PV interneurons, as previously reported [19]. Overall, these data confirm that HFS of Schaffer collateral pathway causes LTP_IE_ in PV interneurons, which is dependent on tetanization, not due to unspecific effects of recording conditions and is associated with a modulation of Kv1.1 channels.

Next, we assessed whether the conditional knock-out of *Rptor* in PV interneurons affected LTP_IE_ since it was reported to be sensitive to the mTORC1 inhibitor rapamycin [19]. In PV-Raptor-Het mice, we found that HFS of Schaffer collaterals failed to induce long-term potentiation of evoked firing in PV mice (n = 13 cells from 5 mice, 91.1% ± 14% of baseline at 10–15 min and 105.5% ± 23.1% of baseline at 25–30 min post-HFS, paired t-tests, p = 0.67 and p = 0.8 respectively, Fig. 3E, F). HFS also failed to alter consistently the first action potential latency (99.66 ± 3.26% of baseline at 10–15 min post-HFS and 98.98 ± 7.33% of baseline at 25–30 min post-HFS, paired t-tests, p = 0.09 and 0.43 respectively, Fig. 3G) and threshold (101.53 ± 1% of baseline at 10–15 min post-HFS and 103.22 ± 1.24% of baseline at 25–30 min post-HFS, paired t-tests, p = 0.15 and 0.02 respectively, Fig. 3H). As in PV-Raptor-WT mice, the absence of HFS stimulation in PV-Raptor-Het mice did not affect PV interneuron evoked firing (n = 5 cells from 3 mice, 85.11% ± 9.6% of baseline at 10–15 min and 88.9% ± 20% of baseline at 25–30 min post-HFS, paired t-tests, p = 0.16 and p = 0.37 respectively, Fig. 3E, F), latency of first action potential (91.86 ± 6.73% of baseline at 10–15 min post-HFS and 91.1 ± 8.35% of baseline at 25–30 min post-HFS, paired t-tests, p = 0.23 and 0.31 respectively, Fig. 3G) or threshold of first action potential (102.66 ± 1.57% of baseline at 10–15 min post-HFS and 99.27 ± 2.27% of baseline at 25–30 min post-HFS, paired t-tests, p = 0.20 and 0.76 respectively, Fig. 3H). These data suggest that conditional heterozygous deletion of *Rptor* in PV interneurons is sufficient to prevent LTP_IE_.

In PV-Raptor-Homo mice, HFS failed to induce a potentiation of evoked firing at 10–15 min but did elicit an increase in firing at 25–30 min post-HFS (n = 14 cells from 5 mice, 115.53% ± 10% of baseline at 10–15 min and 144.9% ± 18.7% of baseline at 25–30 min post-HFS, paired t-tests, p = 0.23 and p = 0.04 respectively, Fig. 3I, J). Similarly, HFS failed to alter latency of the first action potential at 10–15 min but not at 25–30 min post-HFS (97.67 ± 9.64% of baseline at 10–15 min post-HFS, paired t-test, p = 0.4, and 80.95 ± 6.95% of baseline at 25–30 min post-HFS, Shapiro–Wilk test, p = 0.008, Fig. 3K). HFS reduced threshold of the first action potential at 10–15 min but not at 25–30 min post-HFS (102.62 ± 1.28% of baseline at 10–15 min post-HFS and 102.86 ± 1.5% of baseline at 25–30 min post-HFS, paired t-tests, p = 0.04 and p = 0.07 respectively, Fig. 3L). These results suggest that LTP_IE_ is impaired by homozygous deletion of *Rptor*, but that an mTORC1-independent late component of LTP_IE_ remains, as previously reported in experiments using the mTORC1 inhibitor rapamycin [19]. In the absence of HFS, PV interneuron from PV-Raptor-Homo mice did not show change over the same time period in evoked firing (n = 6 cells from 3 mice, 100.3% ± 11% of baseline at 10–15 min and 99.9% ± 20% of baseline at 25–30 min post-HFS, paired t-tests, p = 0.56 and p = 0.72 respectively, Fig. 3I, J), latency to first action potential (90.06 ± 9.35% of baseline at 10–15 min post-HFS, paired t-test, p = 0.64 and 104.66 ± 16.60% of baseline at 25–30 min post-HFS, Shapiro–Wilk test, p = 0.68 respectively, Fig. 3K) and threshold of first action potential (100.15 ± 1.58% of baseline at 10–15 min post-HFS and 100.05 ± 1.6% of baseline at 25–30 min post-HFS, paired t-tests, p = 0.95 and p = 0.98 respectively, Fig. 3L), confirming stable evoked firing over the recording period in these mice also.

Overall, these results show that (i) HFS of Schaffer collaterals induces LTP_IE_ in PV interneurons of PV-Raptor-WT mice, (ii) LTP_IE_ is blocked at both 10–15 min and 25–30 min post-HFS in PV-Raptor-Het mice, and (iii) LTP_IE_ is deficient at 10–15 min but not 25–30 min post-HFS in PV-Raptor-Homo mice (Fig. 3M, 10–15 min post-HFS: One way ANOVA, F (2, 34) = 6 p = 0.003, Tukey’s multiple comparisons test, PV-Raptor-WT vs PV-Raptor-Het, p = 0.004, PV-Raptor-WT vs PV-Raptor-Homo, p = 0.036, PV-Raptor-Het vs PV-Raptor-Homo, p = 0.53; Fig. 3N, 25–30 min post-HFS: One way ANOVA, F(2, 34) = 3 p = 0.04, Tukey’s multiple comparisons test, PV-Raptor-WT vs PV-Raptor-Het, p = 0.03, PV-Raptor-WT vs PV-Raptor-Homo, p = 0.27, PV-Raptor-Het vs. PV-Raptor-Homo, p = 0.48). These findings indicate that the hetero- and homozygous conditional knock-out of *Rptor* in PV INs impairs LTP_IE_, consistent with previous report that this plasticity is sensitive to the mTORC1 inhibitor rapamycin [19].

### Mice with conditional *Rptor* knock-out in PV interneurons show normal contextual fear memory and fear discrimination

**Fig. 4:**
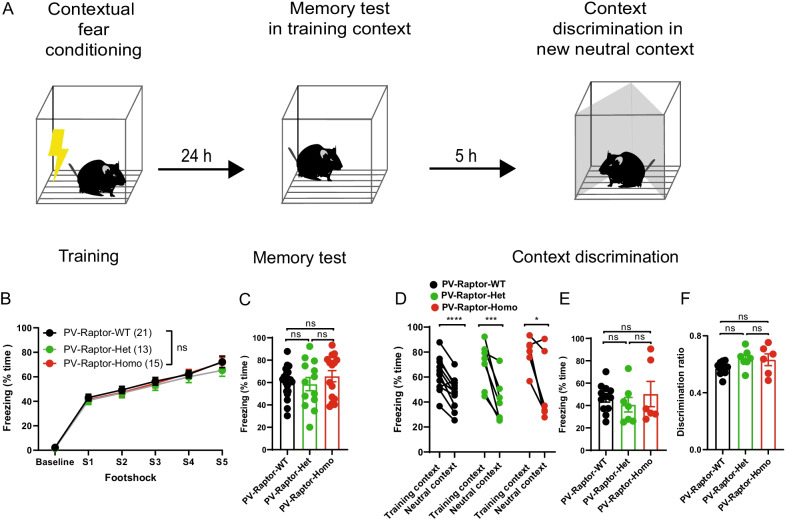
Conditional knock-out of *Rptor* in PV interneurons does not affect contextual fear memory or context discrimination. **A** Diagram of the contextual fear memory and context discrimination protocol. **B** Percentage of time freezing after each foot shock during the training session for PV-Raptor-WT (n = 21), PV-Raptor-Het (n = 13) and PV-Raptor-Homo (n = 15) mice (Baseline: before the first foot shock), indicating similar anxiety level and sensorimotor gating in the three groups. **C** Percentage of time freezing during the long-term memory tests at 24 h in the PV-Raptor-WT, PV-Raptor-Het and PV-Raptor-Homo mice (same mice as in **B**), indicating similar long-term contextual memory in the three groups. **D**–**F** Percentage of time freezing during the contextual discrimination test, relative to the training context (**D**), in the new neutral context (**E**) and discrimination ratio (**F**; amount of freezing in [training context]/[training context + neutral context]) for PV-Raptor-WT (n = 12), PV-Raptor-Het (n = 7) and PV-Raptor-Homo (n = 6) mice, indicating similar context discrimination in the three mice groups. **** *p* < 0.0001, *** p < 0.001, * *p* < 0.05, ns not significant

LTP of PV interneuron excitatory synapses and coherence of PV interneuron firing with CA1 network oscillations are required for contextual fear memory consolidation [35, 36]. Since we found a deficit in LTP_IE_ in PV interneurons with conditional *Rptor* knock-out, next we examined fear memory consolidation and discrimination in these mice. During contextual fear conditioning, PV-Raptor-Het and PV-Raptor-Homo mice showed similar freezing responses to foot shocks relative to PV-Raptor-WT mice (n = 21 PV-Raptor-WT mice, 13 PV-Raptor-Het mice, and 15 PV-Raptor-Homo mice; Two way ANOVA F (2, 46) = 0.358, p = 0.7, Fig. 4A, B), indicating normal anxiety and sensorimotor gating in the mutant mice.

During the long-term memory test in the training context (24 h after conditioning), PV-Raptor-Het and PV-Raptor-Homo mice showed similar freezing responses relative to PV-Raptor-WT mice (One way ANOVA, F (2, 46) = 0.5923 p = 0.5572, Fig. 4A–C), indicating intact long-term contextual memory in the mutant mice. During the context discrimination test in a new neutral context, the three mice groups showed reduced freezing responses relative to the training context, indicating significant contextual discrimination (paired t-tests; n = 12 PV-Raptor-WT mice, p < 0.0001; n = 7 PV-Raptor-Het mice, p = 0.0026; n = 6 PV-Raptor-Homo mice, p = 0.026; Fig. 4D). In the neutral context, PV-Raptor-Het and PV-Raptor-Homo mice showed similar freezing responses relative to PV-Raptor-WT mice (One way ANOVA, F (2, 22) = 0.4621 p = 0.6359, Fig. 4E). Similarly, discrimination ratios to assess context discrimination normalized to the freezing level in the training context were similar in control and mutant mice (One way ANOVA, F (2, 22) = 2.83 p = 0.08, Fig. 4F). These results suggest that long-term contextual fear memory and context discrimination are intact in mice with conditional *Rptor* knock-out in PV INs, and, thus, mTORC1 regulation of firing and long-term potentiation of intrinsic excitability of PV INs may not be necessary for long-term contextual fear memory and context discrimination.

## Discussion

The major results of the present study are, first, that homozygous conditional knock-out of *Rptor* in parvalbumin-expressing cells decreases the level of expression of Raptor, as well as mTORC1 signaling as assessed by immunofluorescence of S6 phosphorylation, in CA1 PV INs (Fig. 1). Second, using whole-cell recordings from CA1 PV INs we found that repetitive firing induced by depolarizing pulses was impaired in mice with either homozygous or heterozygous conditional knock-out of *Rptor*, whereas basic membrane properties and single action potential firing characteristics were unaffected, indicating an impairment in repetitive firing output (Fig. 2). Third, we showed that brief high frequency stimulation of Schaffer collateral synaptic inputs induces LTP_IE_ in PV INs of control mice but failed to do so in mice with either heterozygous or homozygous conditional knock-out of *Rptor* in PV INs, indicating that mTORC1 function is necessary for long-term potentiation of intrinsic excitability (Fig. 3). Fourth, at the behavioral level, we found that mice with homozygous or heterozygous conditional knock-out of *Rptor* showed similar long-term contextual fear memory or contextual fear memory discrimination relative to control mice (Fig. 4). Overall, our results establish a role of mTORC1 in the regulation of repetitive firing and of LTP_IE_ in CA1 PV INs and suggest that mTORC1-regulation of firing and of LTP_IE_ in these interneurons may not be necessary for hippocampus-dependent contextual fear memory and context discrimination.

### Raptor expression and mTORC1 signaling

Raptor constitutes an essential component of the mTORC1 complex [43], whose activation regulates major cellular function such as growth, proliferation and cell metabolism [25], as well as regulation of protein synthesis necessary for synaptic plasticity and memory [27, 44]. Using immunohistochemical assays for Raptor expression and phospho-specific S6 immunohistochemical assay for mTORC1 signaling, we found reduced level of Raptor expression and mTORC1 activity in PV INs from PV-Raptor-Homo mice but not from PV-Raptor-Het mice. However, we found significant impairment in cell firing and LTP_IE_ in PV INs, consistent with some reduction in mTORC1 function in both PV-Raptor-Homo and PV-Raptor-Het mice. Thus, our results suggest that a reduction in mTORC1 function may occur in PV INs with conditional *Rptor* haploinsufficiency which is sufficient to affect mTORC1-dependent firing output and LTP_IE_, but which the Raptor and phospho-S6 immunocytochemical assays are not sensitive enough to detect. Although, S6 phosphorylation is considered a readout of mTORC1 activity [45], other non-mTORC1 intracellular mechanisms also regulate S6 phosphorylation at serine^240/244^ sites, such as PKA-dependent inhibition of the Protein-Phosphatase-1 (PP-1) [46], thus possibly affecting the pS6 assay.

mTORC1 signals to its downstream targets, including S6 phosphorylation, to promote protein synthesis in long-term synaptic plasticity and memory. Indeed, heterozygous knock-out of mTOR in hippocampal pyramidal cells [47] and of *Rptor* in somatostatin interneurons [29] are not sufficient to impair mTORC1-mediated protein synthesis and long-term synaptic plasticity. Thus, homozygous deletions seem necessary to impair mTORC1-mediated protein synthesis, which may appear inconsistent with our results. However, mTORC1 activity represses the local, dendritic mRNA translation of the voltage-gated potassium channel subunit Kv1.1 [40] and reduction in mTORC1 signaling by rapamycin treatment causes the degradation of high affinity HuD target mRNAs, freeing HuD to bind Kv1.1 mRNA and promoting its translation [41]. Thus, mTORC1 regulation of Kv1.1 channel is not via activation of protein synthesis. Our results suggest that heterozygous deletion of *Rptor* is sufficient to reduce mTORC1 signaling involved in regulation of repetitive firing and of Kv1.1 channels during LTP_IE_ in PV IN, in contrast to mTORC1 signaling and p-S6 mediated activation of protein synthesis in synaptic plasticity [29, 47].

mTORC1 downstream signaling involves multiple pathways. Another primary mTORC1 downstream signaling pathway is via phosphorylation of eIF4E-binding proteins (4E-BPs) to activate eIF4E-dependent translation [27]. In addition, via phosphorylation of S6 kinase it targets phosphorylation of S6, but also phosphorylation of eIF4B, inhibition of FMRP signaling, and inhibition of eEF2-kinase [27, 48]. Moreover, mTORC1 controls translation via upregulation of 5' terminal oligopyrimidine (5' TOP) mRNAs that encode components of the translational machinery [27, 49, 50]. We used p-S6 as a readout of mTORC1 signaling and found a deficit in p-S6 signaling in PV-Raptor-Homo but not PV-Raptor-Het mice. However, we observed altered repetitive firing and LTP_IE_ phenotypes in both PV-Raptor-Homo and -Het mice. These results suggest that mTORC1-mediated p-S6 signaling is not associated with the firing and LTP_IE_ phenotypes. Thus, mTORC1 regulation of firing and LTP_IE_ may involve a downstream signaling pathway other than p-S6. Further experiments will be necessary to distinguish possible roles via 4E-BPs, 5' TOP mRNAs, or other targets of S6K, in these mTORC1 mechanisms.

### mTORC1 and PV IN excitability

Our results indicate that impairing mTORC1 function by conditional hetero- and homozygous *Rptor* knock-out selectively decreased firing output of PV INs, without altering their basic membrane properties and single action potential firing characteristics. *Rptor* knock-out in neurons has been associated with numerous morphological abnormalities, such as reduced soma size and dendritic length [51–53], as well as impaired passive and active membrane properties, including input resistance and action potential amplitude [51]. Thus, the reduced cell excitability of PV INs after conditional *Rptor* knock-out could be related to somatic or dendritic morphological changes. Although we did not examine the morphology of recorded cells, the impairment in PV cell firing is unlikely to be due to morphological changes, since we found that basic membrane properties such as resting membrane potential and input resistance, as well as single action potential properties, were intact in PV INs with conditional hetero- or homozygous *Rptor* knock-out. It is important to note that in PV IN conditional knock-out mice, Cre recombination occurs postnatally in hippocampal PV interneurons. Our results corroborate the lack of morphological and membrane properties changes with conditional homozygous *Rptor* knock-out in CA1 somatostatin INs, another mouse model with Cre recombination late in development of interneurons [29]. Interestingly, in contrast to PV INs, *Rptor* knock-out in somatostatin INs is associated with an increase in evoked firing output [29], suggesting that mTORC1 regulates interneuron excitability in a cell type-specific manner.

Fast-spiking interneurons excitability is strongly influenced by Kv1.1-containing potassium channels through regulation of action potential voltage threshold and near-threshold responsiveness [54]. These channels are localized in the soma, dendrites, axon initial segment and synaptic terminals of neurons [55] and their activation dampens neuronal excitability [56]. However, repetitive firing of fast-spiking interneurons is controlled largely by Kv3 potassium channels [57, 58]. Importantly, mTORC1 inhibition with rapamycin increases Kv1.1 expression in dendrites of hippocampal pyramidal neurons [40, 41, 59, 60]. In PV interneurons, as in other neurons, reduction in Kv1.1 lowers the threshold and latency for action potential firing [19, 54, 59, 61, 62]. Our results that the changes in repetitive firing were not associated with any change in threshold or latency of action potentials in PV INs with conditional hetero- or homozygous *Rptor* knock-out, suggest that Kv1 potassium channels may not be the target of mTORC1 regulation to modulate repetitive firing of PV cells. An alternative may be that mTORC1 regulates the expression of Kv3 channels [57], however this remains to be demonstrated.

### mTORC1 and LTP_IE_ in PV INs

Previous work has shown that HFS applied to Schaffer collateral inputs induces a long-term increase in intrinsic excitability of CA1 fast-spiking PV INs in young rats which is prevented by rapamycin treatment, suggesting a role of mTORC1 signaling pathway [19]. However, mTOR signaling can occur via two distinct complexes, mTORC1 that contains Raptor, and mTORC2 that contains Rictor [27]. Hippocampal long-term synaptic plasticity and memory involve both mTORC1 [47] and mTORC2 [63] signaling. Although rapamycin is considered a more effective mTORC1 inhibitor [32], prolonged treatment [33] or higher concentration [34] of rapamycin also inhibits mTORC2. Thus, sensitivity to rapamycin treatment does not necessarily indicate mTORC1 implication. Our findings that HFS induced long-term increase of PV INs intrinsic excitability in hippocampal slices from control mice, but failed to do so in mice with hetero- or homozygous conditional *Rptor* knock-out, clearly indicate that mTORC1 activity is required for LTP_IE_ in PV INs, extending previous findings obtained with rapamycin treatment [19].

Our results show that LTP_IE_ is completely blocked in PV INs with heterozygous *Rptor* knock-out at 10–15 min and 25–30 min time points after HFS, but only at 10–15 min after induction in PV INs with homozygous *Rptor* knock-out, indicating a residual late component of LTP_IE_ after complete *Rptor* knock-out. Our results share some similarities with previous findings that treatment of hippocampal slices with rapamycin suppressed LTP_IE_ at 10–15 min after induction but did not block completely LTP_IE_ at later times (25–30 min) [19]. Thus, our results indicate, first, that *Rptor* haploinsufficiency is sufficient to completely prevent LTP_IE_ in PV INs, clearly showing a requirement for mTORC1 activity in LTP_IE_ in PV INs. Second, they indicate that a residual component of LTP_IE_ is present at later times in mice with full knock-out of *Rptor*. Further experiments will be necessary to identify the mechanisms possibly involved.

In CA1 fast-spiking PV INs, brief repetitive stimulation of Schaffer collaterals induces a rapamycin-sensitive LTP_IE_ which is mediated by synaptic activation of mGluR5 [19]. Moreover, LTP_IE_ involves a down-regulation of Kv1.1 channel activity since pharmacological blockers of Kv1.1 mimick LTP_IE_ and occlude further induction of LTP_IE_ [19]. Thus, our findings that LTP_IE_ in PV INs is associated with a decrease in the latency and threshold of evoked action potentials, which is consistent with previous findings [19], and that LTP_IE_ is impaired by conditional knock-out of *Rptor* in PV cells, provide a link between mTORC1 activation and regulation of Kv1.1 channel activity during LTP_IE_. These findings of mTORC1 requirement in LTP_IE_ are consistent with previous evidence that mTORC1 activity regulates negatively Kv1.1 channel expression and activity in pyramidal cell dendrites [40, 41, 59]. Our observations, thus, provide functional evidence of an mTORC1 regulation of Kv1.1 channel function during activity-dependent long-term plasticity of PV interneuron intrinsic excitability.

### mTORC1 and hippocampal memory

Multiple lines of evidence indicate an important role of PV IN activity and synaptic plasticity in hippocampus-dependent memory function. Long-term structural plasticity of excitatory and inhibitory synapses of PV INs contributes to contextual fear learning and memory consolidation, as well as maze navigation learning [64]. Pharmacogenetic inhibition of PV INs prevents contextual fear conditioning-induced changes in network oscillations and impairs fear memory consolidation [35]. In addition, genetic deletion of γCaMKII in PV INs prevents LTP at their excitatory input synapses from Schaffer collaterals and impairs fear memory consolidation [36]. Although there is no evidence of a role of mTORC1 in these synaptic plasticity mechanisms, our observations that contextual fear memory and context discrimination are intact in mice with conditional *Rptor* knock-out in PV cells, indicate that mTORC1 signaling is not involved in the roles of these plasticity mechanisms of PV INs in hippocampus-dependent memory tasks. These conclusions are in contrast with evidence in hippocampal principal cells and in somatostatin interneurons that mTORC1 signaling plays a critical role in long-term synaptic plasticity and in hippocampus-dependent learning and memory consolidation [29, 47], pointing to cell type-specific mTORC1 mechanisms in long-term synaptic plasticity and memory consolidation.

Moreover, our findings that mice with conditional *Rptor* knock-out in PV cells show deficits in LTP_IE_ and intact hippocampal-dependent contextual fear memory and context discrimination, indicate that mTORC1-dependent LTP_IE_ in PV INs may not be necessary for these hippocampus-dependent memory tasks. This is in contrast with excitatory cells where plasticity of intrinsic excitability, also expressed as a change in action potential firing, plays an important role in memory allocation, consolidation, and updating [21–23]. Given that LTP_IE_ promotes PV INs firing in the gamma range and facilitates their recruitment by pyramidal cells [19] which may favor synchronization of pyramidal cells activity and generation of network oscillations [65], our results raise the question of when is mTORC1-dependent LTP_IE_ in PV INs critical for hippocampus-dependent learning and memory consolidation? Impairment of inhibition by hippocampal PV INs results in impaired spatial working memory and intact spatial learning and spatial reference memory [66]. Thus, LTP_IE_ and regulation of PV INs firing in the generation of network oscillations [65] could be important for learning during spatial navigation [67–69]. Interestingly, increased mTORC1 activity in mice with conditional heterozygous knock-out of *Tsc1* in Nkx2.1 expressing interneurons, which include somatostatin and parvalbumin interneurons, impaired hippocampus-dependent long-term spatial working memory but not spatial reference memory [37]. Thus, mice with conditional *Rptor* knock-out in PV INs could be useful to determine if mTORC1-mediated LTP_IE_ is implicated in long-term spatial working memory.

Interneuron type-specific mTORC1 function is important in pathological conditions. The eukaryotic translation initiation factor 4E-binding protein 2 (4E-BP2) is a translational repressor downstream of mTORC1. Genetic ablation of 4E-BP2 in inhibitory but not excitatory neurons causes an increase in the susceptibility to pentylenetetrazole-induced seizures [70]. Moreover, mice lacking 4E-BP2 in parvalbumin, but not in somatostatin or vasoactive intestinal peptide-expressing (VIP) inhibitory neurons exhibit a lowered threshold for seizure induction and reduced number of parvalbumin neurons [70]. Thus, increased mTORC1-dependent translation in parvalbumin neurons is implicated in the pathophysiology of epilepsy [70]. Such a role is consistent with PV IN dysfunction contributing to epileptiform discharges and abnormalities in oscillatory rhythms, network synchrony, and memory in human amyloid precursor protein (hAPP) mouse model of Alzheimer disease [71]. In addition, deletion of 4E-BP2 in GABAergic inhibitory neurons results in impairments in social interaction and vocal communication [72]. Thus, mTORC1 signaling via 4E-BP2 has an inhibitory cell-specific role in engendering autism related behaviors [72]. These findings are consistent with a loss of hippocampal PV INs, impaired perisomatic inhibition, gamma and sharp wave ripples activity, as well as spatial discrimination, in the *Cntnap2* mouse model of autism spectrum disorder [73]. Thus, an implication of mTORC1-mediated LTP_IE_ in PV INs in interneuron-specific pathological conditions would be important to investigate.

In conclusion, we found that mTORC1 activity regulates CA1 PV IN repetitive firing and LTP_IE_ but may not be necessary for consolidation of long-term contextual fear memory and context discrimination. Thus, mTORC1 plays cell-specific roles in synaptic plasticity of hippocampal inhibitory interneurons that are differentially involved in hippocampus-dependent learning and memory.

### Limitations

Some limitations should be considered when interpreting our results. First, although our interpretations discussed in the study are concordant with impaired mTORC1 activity at least in CA1 PV INs of mice with conditional homozygous knock-out of *Rptor* in PV cells, functional alterations of PV INs in other hippocampal and neocortical regions could also influence the behavioral tasks in our study. Although we tested a behavioral task known to engage the hippocampus (contextual fear memory), a region-specific deletion approach would be helpful to confirm the role of CA1 PV INs. Second, we used immunohistochemical assays for Raptor expression and phospho-specific S6 immunohistochemical assay for mTORC1 signaling and we could detect a reduction of Raptor protein expression level and mTORC1 activity in PV INs from mice with conditional homozygous *Rptor* knock-out but not in mice with heterozygous *Rptor* knock-out. However, we did find phenotypes in electrophysiological experiments in PV INS of both hetero- and homozygous *Rptor* knock-out mice, suggesting a reduction in Raptor expression level with *Rptor* haploinsufficiency. A more sensitive assay, such as quantitative single cell PCR would be necessary to confirm the effective knock-down of *Rptor* in mice with heterozygous deletion. Third, previous studies focusing on mTORC1 in the regulation of synaptic transmission, revealed that reducing mTORC1 activity with rapamycin or by knocking out Raptor, decreased the efficacy of excitatory transmission via pre- and postsynaptic mechanisms in hippocampal excitatory neurons [51, 74, 75]. Thus, if similar mechanisms occur in PV INs, excitatory synaptic inputs in PV INs could be reduced in mice with conditional hetero- or homozygous *Rptor* knock-out, which may reduce the postsynaptic efficacy of HFS to activate mGluR5 and induce LTP_IE_. Thus, characterization of synaptic transmission in conditional *Rptor* knock-out mice would be necessary to rule out such changes. However, in our experimental protocol, the strength of synaptic stimulation was adjusted to elicit similar EPSPs prior to induction of LTP_IE_ and control for putative differences in presynaptic release in the different genotypes. Fourth, we found that conditional hetero- or homozygous *Rptor* knock-out mice showed altered repetitive firing and a block of LTP-IE, indicating a role of mTORC1. However, we did not test in parallel the possible role of mTORC2. Similar experiments using conditional Rictor knock-out mice would be useful to assess this possibility. Fifth, our conclusion that mTORC1 function in PV interneurons may not be necessary for contextual fear memory and context discrimination is based on a single type of behavioral experiments (contextual fear memory) in which we found no difference between wild-type and transgenic mice. Thus, additional behavioral verifications with other hippocampus-dependent tasks would be necessary to confirm our conclusion. Finally, a last limitation to consider is that our whole cell recordings were obtained from cells in slices that came from a smaller number of transgenic mice. However, all whole cell recordings were obtained from a single cell per slice which are considered independent experiments. Nonetheless, additional experiments in a larger number of mice would clarify this issue.

## Data Availability

The datasets used and/or analyzed during the current study are available from the corresponding author on reasonable request.

## Abbreviations

aCSF: Artificial cerebrospinal fluid
AP: Action potential
AHP: After hyperpolarization
CaMKII: Ca2+/calmodulin-dependent protein kinase II
CFC: Contextual fear conditioning
ERK: Extracellular signal-regulated kinase
FS-PV INs: Fast-spiking parvalbumin-expressing interneurons
HFS: High frequency stimulation
LTD: Long-term depression
LTD_IE_: Long-term depression of intrinsic excitability
LTP: Long-term potentiation
LTP_IE_: Long-term potentiation of intrinsic excitability
mGluR5: Type 5 metabotropic glutamate receptors
mTORC1: Mammalian target of rapamycin complex 1
PI3K: Phosphoinositide 3-kinase
PKA: Protein kinase A
PKC: Protein kinase C
PV INs: Parvalbumin-expressing interneurons
Raptor: Regulatory-Associated Protein of mTOR
Rin: Input resistance
RMP: Resting membrane potential
SOM INs: Somatostatin interneurons
TSC2: Tuberous Sclerosis Complex 2
γCaMKII: Gamma Ca2+/calmodulin-dependent protein kinase II
